# Comparison of two cannulation methods for assessment of intracavernosal pressure in a rat model

**DOI:** 10.1371/journal.pone.0193543

**Published:** 2018-02-27

**Authors:** Shankun Zhao, Ran Kang, Tuo Deng, Lianmin Luo, Jiamin Wang, Ermao Li, Jintai Luo, Luhao Liu, ShawPong Wan, Zhigang Zhao

**Affiliations:** 1 Department of Urology & Andrology, Minimally Invasive Surgery Center, Guangdong Provincial Key Laboratory of Urology, The First Affiliated Hospital of Guangzhou Medical University. Guangzhou, Guangdong, China; 2 Department of Urology, The First Affiliated Hospital of Nanhua University. Hengyang, Hunan, China; University of Southampton, UNITED KINGDOM

## Abstract

Intracavernous pressure (ICP) measurement is a well-established technique for assessing the erectile function, which was performed by cannulating either crus or shaft of the penis. However, there are no studies concerning the experimental performance of the two cannulation sites yet. The aim of this study was to compare the measuring outcomes using two different cannulation sites. To validate the capacity of our study, both normal and the castration-induced erectile dysfunction rat models were conducted. Fifty adult male Sprague-Dawley rats were randomized equally into two groups: an intact group and a castration group. Five rats from each group firstly underwent different stimulation parameters to detect the optimal erectile responses. The residual rats in each group were further assigned into two subgroups (n = 10 per subgroup) according to two different cannulation sites (crus or shaft of the corpus cavernosum). The ICP values were compared between groups after different interventions. The optimal parameters for mean maximum ICP were recorded at 2.5V and a frequency of 15 Hz. The rats under the two different cannulation sites tended to show similar ICP values in both the intact and the castration groups. However, the success rate in monitoring ICP was significantly higher in the groups cannulating into the shaft of the penis compared to the crus (100% *vs*. 70%; *P* = 0.02). Our data suggested that the method of cannulation into the penile shaft could serve as a better alternative for the ICP measurement in rats.

## Introduction

The current knowledge of the physiology of penile erection and the investigative pathophysiology of erectile dysfunction (ED) critically depends on animal models [1, 2]. Numbers of different in vivo approaches have been used to investigate the erectile function in experimental animal models. These include but are not limited to penile plethysmography [3], screening tests of sexual behavior [4, 5], apomorphine-induced erectile response [6], and dynamic cavernosography [7]. However, none of these assays is ideal for the neurophysiological evaluation of erection because of lack of accuracy and reproducibility. Telemetric assessment of intracavernosal pressure is a novel technique for erectile evaluation, which is performed with a pressure sensor surgically implanted in the corpus cavernosum [8]. It can monitor both the qualitative and the quantitative parameters of the erections in a conscious animal. However, this technique was reported to be limited by the high failure rate due to the frequent obstruction of the catheter tip [9].

Intracavernosal pressure (ICP) measurement after electrical stimulation (ES) of the major pelvic ganglion (MPG) or cavernous nerve (CN) elicited penile erection in anesthetized animals is a detection technique that was originally developed by Quinlan et al [10]. This standardized method was well established and has been documented as an objective and accurate biometric on the evaluation of erectile function [11]. It provides the most reliable and quantitative response of the penis to peripheral and central nervous system neural activation. Accordingly, it contributes greatly to the assessment of a normal or ED experimental animal model as well as the development of potential therapeutic strategies [12–14].

To monitor the ICP, a needle is typically inserted into either the crura [15, 16] or the shaft of the penis [1, 17] and connect to a pressure transducer. Our laboratory routinely performs the ICP measurement, and our experience is that cannulation of the crura occasionally fails because of tumescence. Therefore, we turned to measure the penile pressure in the body of the penis and achieved satisfactory results in our previous study [18, 19]. As far as we know, there are no studies concerning the experimental performances between two cannulation sites (the penile crus and the penile shaft) yet. Therefore, the current study aimed to compare the measuring outcomes using two different cannulation sites of the corpus cavernosum. To validate the capacity of our study, both normal and the castration-induced ED rat models were conducted.

## Materials and methods

### Ethical approval and animal preparation

Fifty 12-week-old male Sprague-Dawley rats, weighing 320–360g, were obtained from Guangdong Medical Laboratory Animal Center (Guangzhou, China) and randomly divided into two groups (25 rats/group): the intact group and the castration group. Random numbers were generated using the standard = RAND() function in Microsoft Excel. Surgical castration was performed using the method described by Pongkan et al [20]. Briefly, rats under anesthesia, a 1-cm midline incision was made in the scrotum and both testicles were removed after ligation and transection of the spermatic cords. Ampicillin was injected subcutaneously for 3 days after bilateral orchiectomy. Androgen deprivation by castration has been known to consistently and markedly diminish erectile function [21], which has been used to establish an ED animal model [22]. The choice of castration-induced ED model in this experiment was mainly due to its ease of setup.

The rats were maintained in pathogen-free conditions in the Laboratory Animal Center of the State Key Laboratory of Respiratory Disease, the First Affiliated Hospital of Guangzhou Medical University. The animals were kept in plastic cages (5 animals per cage) and maintained in an environmentally controlled room (temperature 24°C, relative humidity 60%, and 12-hour light/dark cycle) with free access to fresh solid pellet diet and water. At the end of the pressure recordings, the animals were sacrificed by an anaesthetic overdose intraperitoneal administration of pentobarbital. These protocols were approved by the Ethics Committee on Animal Experimentation of the First Affiliated Hospital of Guangzhou Medical University (approval number 2014–019). A completed ARRIVE guidelines checklist is included in S1 Table.

Four weeks after castration, the body weight of each animal was recorded. The intact animals were similarly weighed at this time. Within each group, five of the 25 rats were randomly selected to undergo different stimulation parameters to detect optimal erectile responses. Once the stimulation parameters were established, the residual rats were randomly assigned into four experimental groups with ten each: Group A: intact rats were performed under cannulation of the penile crus for ICP measurement; Group B: intact rats were subjected to cannulate into the shaft of the penis; Group C and D were castrated rats assigned to the methods of cannulation of the penile crus and the penile shaft, respectively.

### Different voltages and frequencies on ES parameters

For study convenience, 5 of the 25 rats in each group were submitted to ES at the parameters that resulted in the optimal erectile responses. The ES equipment was composed of a bipolar hook electrode and an integrated data acquisition system (MP 150A-CE; Biopac, Santa Barbara, CA). All data were analyzed using the AcqKnowledge software, version 4.2.1 (Biopac System Inc). The application of different voltages and frequencies were used in the current protocol to achieve a significant and consistent erectile response. Under anaesthetic (pentobarbital, 45 mg/kg), rats were first subjected to sequential stimulation strengths ranging from 1 to 10 volts at a frequency of 15 Hz with a square pulse wave form and a duration of 5 milliseconds for 60 seconds. The optimal voltage parameter according to the voltage response curves was logged. Next, the effect of different frequencies (10, 15 and 20 Hz) at the selected voltage that resulted in the highest erectile response was assessed. Three ESs were replicated at intervals of 5 min. After the optimal stimulation parameters were established, the ICP was monitored by different cannulation methods in the residual rats.

### The method of cannulation into the crus of penis for ICP measurement

#### Mean arterial pressure (MAP) measurement

**Fig 1:**
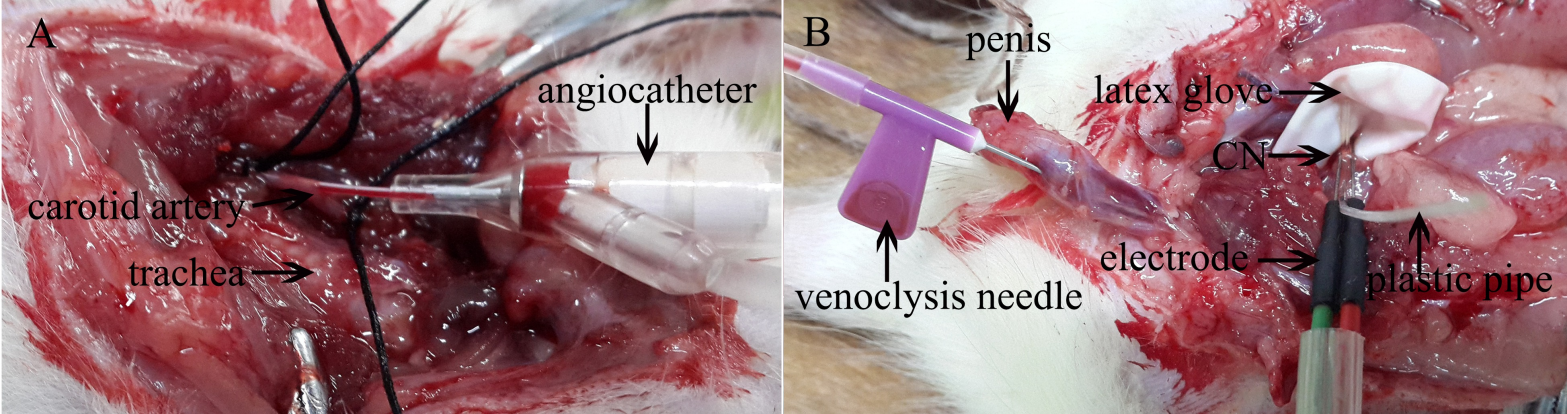
Mean arterial pressure (MAP) and intracavernous pressure (ICP) measurement. (A) The right internal carotid artery was detached and intubated with a 24-gauge angiocatheter to measure MAP. (B) Pulse stimulator was connected to a stainless steel bipolar hook electrode positioned on the cavernous nerves (CN). The parallel hooks were kept apart by an insulator. A piece of a latex glove was used to isolate the distal ends of the electrical stimulators. ICP was recorded with a 24-gauge venoclysis needle inserted into the corpora cavernosum and connected to a pressure transducer.

Continuous monitoring of MAP was performed using the same method introduced in the previous study [23] with some refinements. Briefly, the right carotid artery was exposed and the cephalad end of the carotid artery was ligated by a 4–0 silk suture. This would prevent obscuring of the visual field from the leakage of blood. Next, a slipknot with a silk suture was tied around the proximal end carotid artery. A 24-G angiocatheter filled with heparin and connected to the pressure transducer was then inserted caudally into the carotid artery (Fig 1A). Afterwards, the slipknot suture was tightened to prevent the sensor needle falling out of the vessel. The ratio between the ICP and MAP was calculated to normalize the variations in systemic blood pressure.

#### Exposure of the CN and fixation of the electrode

**Fig 2:**
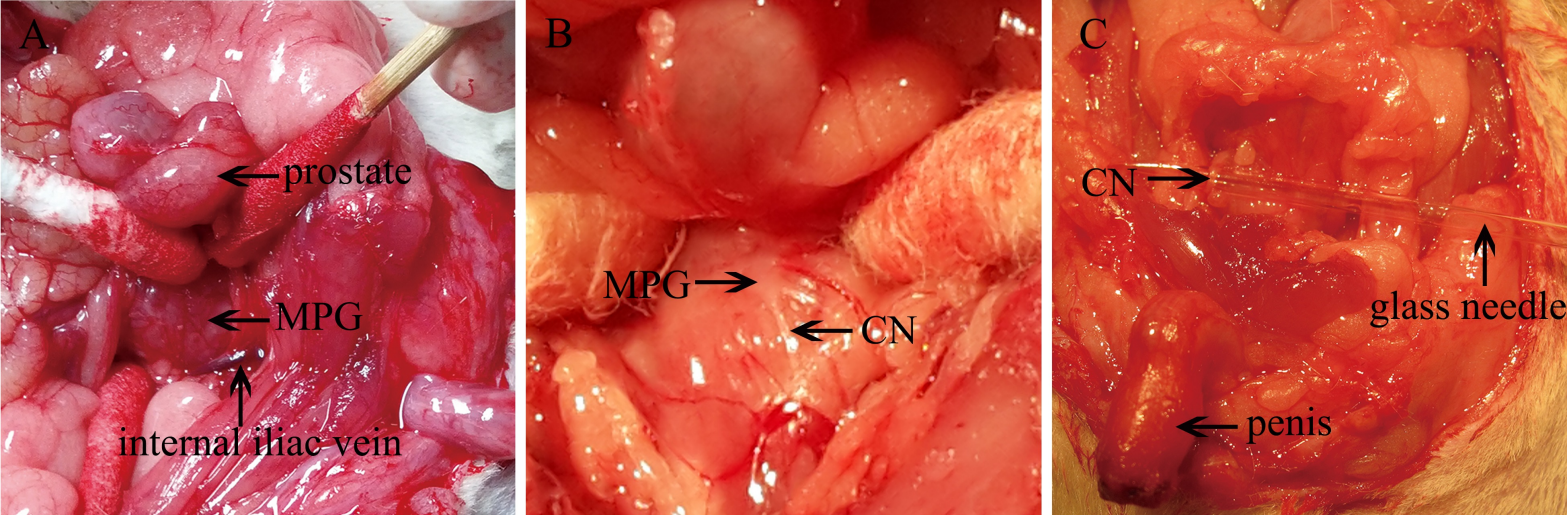
Exposure of the major pelvic ganglion (MPG) and the cavernosal nerve (CN). (A) The MPG can be found next to the internal iliac vein. (B) The exposed MPG and the CN. (C) The CN was isolated using a glass needle.

The procedure for ICP measurement was based on the technique described by Rehman et al [24]. By retracting the intestine, the seminal vesicles, and the bladder laterally, the fascial layer covering the prostate could be exposed posteriorly simply using cotton swabs. We had noted that the prostate gland was usually atrophic in the castrated rat; it was thus often difficult to identify. The star-like MPG, located posterolaterally and symmetrically to the dorsal lobe of the prostate, was visualized. At this time, the internal iliac vein could be seen directly adjacent to the dorsal lobe of the prostate, following its curvature. This is an anatomic landmark we use for identifying the MPG (Fig 2A). The MPG divides into three major branches. The largest that runs along the surface of the membranous urethra is the CN (Fig 2B). The pelvic nerve is situated inferior to the MPG. And the proximal branch that disperses along the intraabdominal position is the hypogastric nerve. We preferred to do the isolation of the CN using a glass needle (Fig 2C). Next, a tiny stainless steel bipolar hook electrode with a diameter of 0.3 millimeters was placed around the CN, 2~4 millimeters distal to the MPG, for ES. The positive electrode (red cover) was placed proximal to the MPG and the negative electrode (green cover) at the distal end. To prevent shorting circuits, the parallel hooks of the electric stimulator were separated from each other by an insulator with a one-millimeter diameter. In addition, we recommended encasing a piece of latex glove to isolate the ends of the electrical stimulators in order to prevent the ES of the surrounding tissues (Fig 1B).

#### Cannulated the corpus cavernosum

**Fig 3:**
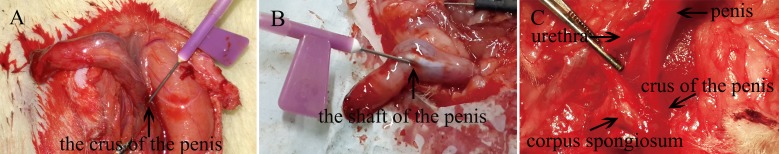
Macroscopic images show different cannulation site in the corpus cavernosum. (A) Cannulating into the crus of the penis. (B) Cannulating into the penile shaft. (C) Anatomic relationships between the crus of the penis, the urethra, and the corpus spongiosum.

After the bipolar electrodes were in place, the penis was degloved and the penile ischiocavernous muscle was divided into one side by blunt dissection. This allowed entry into the underlying tunica albuginea of the crus of the corpus cavernosum. A 24-gauge venoclysis needle was inserted into this side of crus for the ICP measurement (Fig 3A). 50 microliters of heparinized saline solution (250 U/ ml) were injected into the corpora cavernosum with a syringe through the T-shaped connection pipe to prevent clotting of the needle tip. As illustrated in S1 Fig, blood reflux in the piping system after the venoclysis needle was inserted into the corpus cavernosum was an important sign of successful cannulation, and we used this to judge whether the channel between the corpora cavernosum and the pressure transducer was successfully established.

#### Monitoring ICP

Basal ICP was first assessed using the analysis software of AcqKnowledge 4.2.1. The neurostimulation of the CN was next performed and the changes of ICP were recorded. The mean maximum ICP and the total ICP of the tumescence determined by the AUC from the beginning to the end of the CN stimulation (60 seconds) were recorded. The ES was always done in triplets with a 5-minute interval between the subsequent stimulations to ensure stable activity in every rat.

### The method of cannulation of the penile shaft

The surgical protocol for this method was similar to the method of the cannulation into the penile crus. It was performed at the mid-shaft and along the axis of the corpus cavernosal body (approximately 10mm in the distal of the base of the penis) (Figs 1B and 3B). In brief, the overlying skin and fascia surrounding the penis were removed by sharp dissection to expose the penile shaft. Next, a 24-gauge venoclysis needle was inserted into the midpoint of the corpus cavernosum, aiming toward the crus. In this method, the needle and the penile shaft formed a 10-degree angle and allowed a longer length of cannulation, approximately 6–8mm. After the cannulation, the ICP was measured with a pressure transducer connected to a data acquisition system (S1 Video). Also, we have deposited our laboratory protocols of the overall procedure of ICP measurement in protocols.io (S2 Table), please access at dx.doi.org/10.17504/protocols.io.kwdcxa6 for more detail.

### Statistical analysis

SSPS 20.0 software (SPSS, Chicago, IL, USA) was used to analyze the data. The results were expressed as mean ± standard deviation. The ICP examination was performed by the same investigator who was blinded to the grouping of the animals. In addition, raw data were collected and analyzed by two independent blinded observers. Different voltages and frequencies on erectile function parameters were compared by using one-way analysis. Differences amongst body weight, testosterone detected in serum, and ICP values were assessed by Student’s *t-*test. A Fisher's exact test was applied to measure the success rate. Difference with *P* < 0.05 was considered statistically significant.

## Results

### Body weight evaluation

The initial body weight of the intact and the castrated groups did not show any significant difference (*P* > 0.05). At four weeks, the castrated rats had significantly lower body weight than the intact rats (*P* < 0.05 for all) (S3 Table).

### Stimulation parameters studies

**Fig 4:**
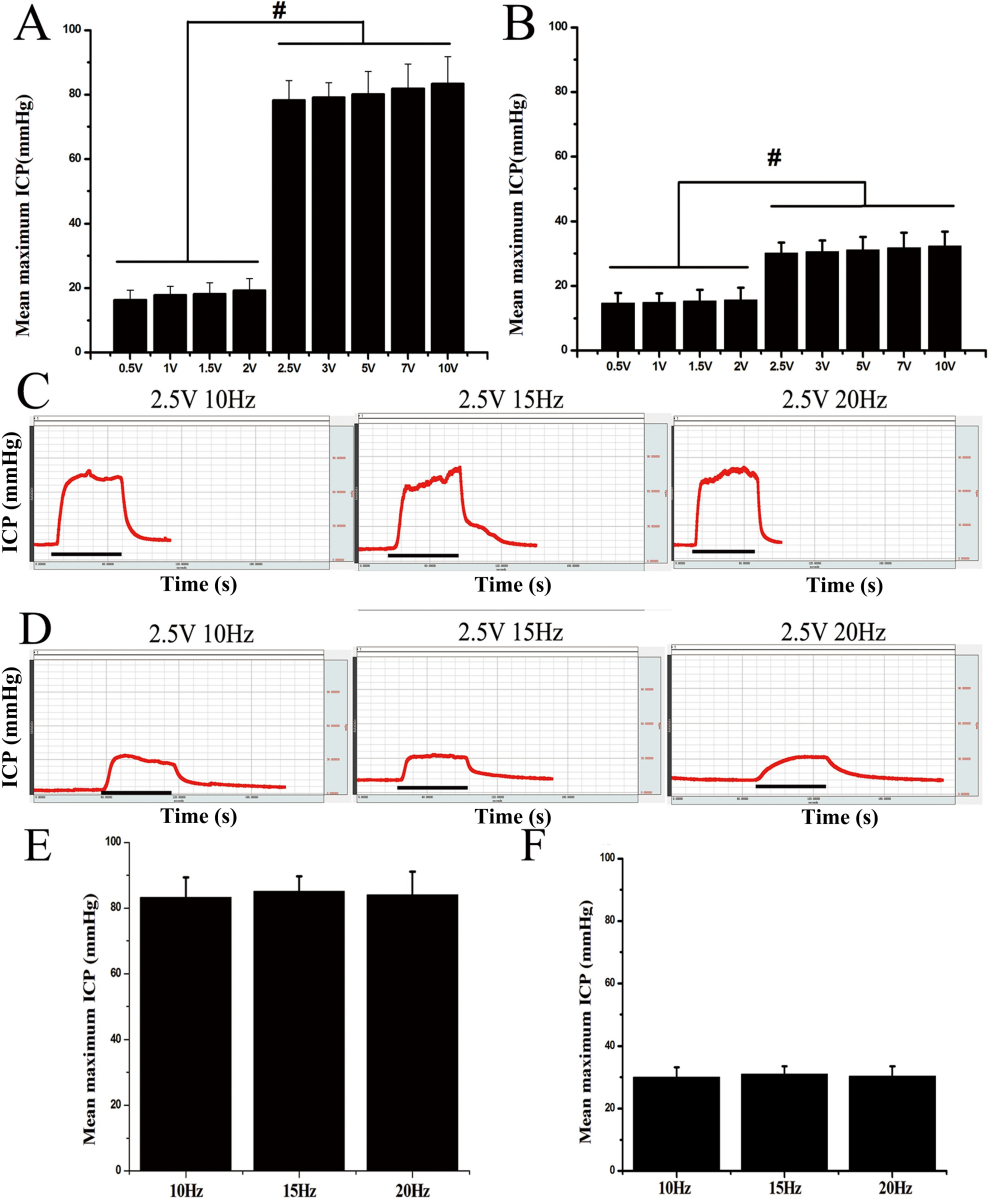
Effect of different voltages and frequencies on the erectile function parameters. (A, B): Quantitative analyses of erectile function at different voltages (1–10 V) of electrical stimulation induced in ICP in the intact group (left) and the castration group (right). (C, D): Representative ICP changes at different frequencies (10, 15 and 20 Hz) in the intact group (left) and the castration group (right). The x-axis depicted seconds and the Y-axis represented changes in ICP, black bars under the x-axis indicated a 60-second period of stimulation. (E, F) Quantitative analyses of mean maximum ICP at different frequencies (10, 15 and 20 Hz) in the intact group (left) and the castration group (right). These results were compiled from 5 of 25 animals in each of the intact group and the castration group. Each bar depicts the mean ± SEM, ^#^*P* < 0.01.

The mean maximum ICP was found to be significantly greater in both the intact and the castrated groups when above 2.5 volts were used (*P* < 0.01, for all) (Fig 4A and 4B). The erectile response could not be elicited under 2.5 V. In addition, the ICP values did not differ significantly over 2.5V. Subsequently, different frequencies (10, 15 and 20 Hz) at a voltage of 2.5 V were tested (Fig 4C and 4D). As illustrated in Fig 4E and 4F, the mean maximum ICP showed a slight but not statistically significant increase at 15 Hz when compared to other frequencies. Thus, these data indicated that 2.5V at 15Hz frequency may exert the optimal effect on the erectile response of the rat. Based on these findings, these ES parameters were used throughout the study.

### Experimental performance between two cannulation sites for ICP measurement

**Fig 5:**
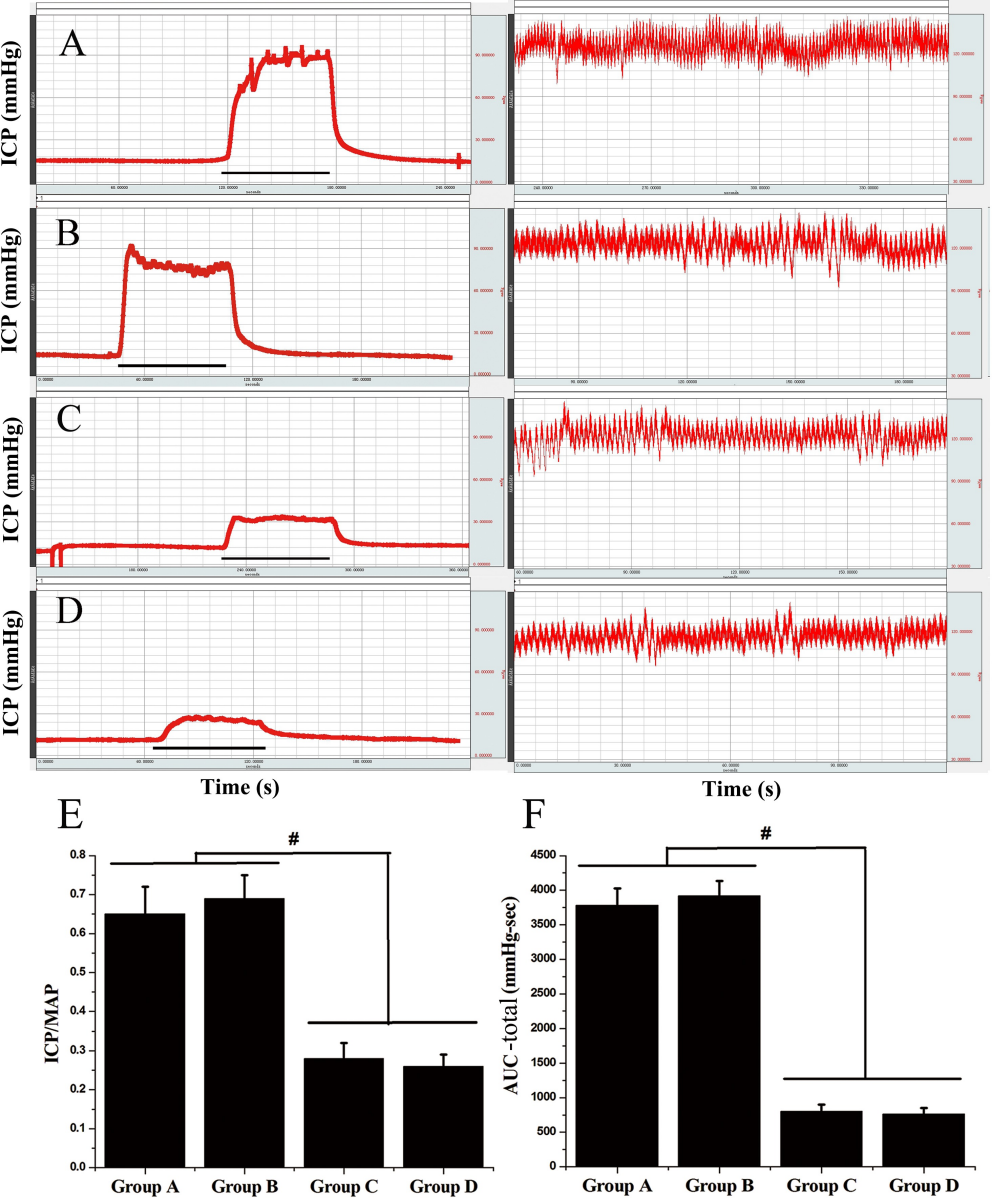
Changes of ICP, MAP, ICP/MAP and the area under the curve (AUC) of each group. (A-D): Biopac physiograph displayed the ICP (left) and MAP (right) of representative rats in Group A-D. The x-axis depicted seconds and the horizontal bar represented one electrical stimulus lasting 60 seconds. The Y-axis represented changes in ICP or MAP. (E, F): Statistical chart of ICP/MAP ratio and total AUC in each group (n = 8, 10, 6, 10, respectively). Bar graphs represent means±SD, ^#^*P* <0.01. Group A = intact rats under the method of cannulation into the crus of the penis; Group B = intact rats cannulating into the penile shaft; Group C = castrated rats with penile crus cannulation; Group D = castrated rats with penile shaft cannulation.

In this study, six rats (30%) failed the ICP measurement using the method of cannulation into the crus of the penis (four in Group A and two in Group C). The investigation results of sensor needle insertion with two methods are shown in Table 1. In four subjects, the needle slipped out of the crus of the penis during the penile tumescence. In the other two, there were great variations in the ICP values when repetition, mainly because of blood leakage. They were thus excluded from the final statistical analysis. Representative samples of ICP and MAP recordings were displayed in Fig 5A–5D. As displayed in Fig 5E and 5F, all castrated rats showed statistically lower ICP/MAP ratio and total AUC than the intact rats (*P* < 0.05, for all), indicating that the rat model with ED had been established.

**Table 1:** Investigation results of sensor needle insertion with two cannulation methods.

	Modified technique (n = 20)		Conventional technique (n = 20)		*P* value
	Intact	Castration	Intact	Castration	
Needle dislodgment	0	0	3	1	–
Blood leakage	0	0	1	1	–
Total success rate (%)	100% (20/20) 70% (14/20)				0.02

Comparisons between two groups using a Fisher's exact test.

The changes in the ICP, including baseline ICP, mean maximum ICP, MAP, ICP/MAP ratio and total AUC were illustrated in Table 2. In the intact group, there were no significant differences between the method of cannulation into the penile crus (Group A) and the shaft of the penis (Group B) (*P* > 0.05, for all). Similarly, no significant differences in these ICP parameters were found between Group C and Group D in castrated rats (*P* > 0.05, for all). However, the method using cannulation site of the penile shaft acted more stable in cannulating the corpus cavernosum than that of the penile crus. It was completed successfully in all of the 20 rats (100%); whereas in the method of cannulation into the crus of the penis, only 14 out of 20 (70%) were completed. There was a significant difference in the success rates between the two methods (*P* = 0.02). All raw research data are shown in S1 File.

**Table 2:** ICP and MAP indexes of rats in each experimental group.

Group	n	Baseline ICP	Mean maximum ICP	MAP	ICP/MAP	AUC-total
		(mmHg)	(mmHg)	(mmHg)		(mmHg-sec)
intact group						
Group A	8	17.0±1.93	81.7±8.94	125.6±4.69	0.65±0.07	3806±230.8
Group B	10	16.6±2.67	86.2±8.02	121.9±5.88	0.70±0.06	3912±192.5
*t*		0.355	-1.112	1.457	-1.736	-1.072
*P*		0.727	0.283	0.164	0.102	0.300
castration group						
Group C	6	16.2±2.93	32.5±3.93	116.8±7.49	0.28±0.05	804±85.5
Group D	10	15.5±2.37	29.8±3.32	113.7±9.63	0.26±0.04	778±74.4
*t*		0.500	1.47	0.680	0.655	0.654
*P*		0.625	0.164	0.508	0.523	0.524

Values are expressed as the mean ± SEM. Comparisons between two groups using a Student’s *t-*test.

Group A, intact rats under the method of cannulation into the penile crus; Group B, intact rats under the method of cannulation into the penile shaft; Group C, castrated rats with penile crus cannulation; Group D, castrated rats with penile shaft cannulation. ICP = intracavernosal pressure, MAP = mean arterial pressure; AUC = area under the curve, total ICP from the beginning of cavernous nerve stimulation to the stimulus termination (60s).

## Discussion

Most of the present knowledge concerning penile hemodynamics and erection resulted from animal models [25]. Studies showed that the morphology of erectile tissue and the distribution of nerves in rat corpus cavernosum are similar with humans [10, 26]. For in vivo evaluation of erectile function in rats, most researchers adopted electrical field stimulation induced ICP changes [27, 28]. It was demonstrated that ES of the CN could mimic the integrated electrophysiological erection [29]. Commonly, ICP was monitored by inserting a tube into the body or the crus of the corpus cavernosum. Although both cannulation sites are routinely applied in the ICP measurement, few experimental studies have been conducted to investigate the validity of two different cannulating methods. As the application of ICP measurement expanding, the proper conduct of the cannulation approach is needed.

Based on this study, our results indicated that there was no significant difference between the ICP values from the two cannulation approaches. However, the success rate in monitoring ICP was significantly higher in the group using the method of cannulation into the shaft of penis compared to the group cannulating into the penile crus in a rat model. It seems that the use of the method cannulating into the penile shaft might be superior to cannulate into the penile crus when performing ICP measurement.

In a rat model, the crura of the corpus cavernosum diverge and taper at the level of the bulb of corpus spongiosum. The crura are covered inferiorly by the bulbospongiosum and the bulbocavernosum muscles, and superiorly by the pubic arch. The exposed area of the crus here is narrow. Thus, it is hard to gauge and insert the sensor needle accurately (Fig 3C). Due to the shallow depth of the cannulation (3–5mm), the sensor needle often fell out of the tunica albuginea during tumescence; there also seemed to be more blood leaking from the cannulation site. However, deeper cannulation might result in perforation through the crura into the corpus spongiosum by the needle. In such an event, the recorded pressure would be the spongiosal pressure rather than the cavernosal pressure. In this experiment, we had six rats fail in both intact and castration groups with the method of cannulation into the penile crus. Adequate ICP data were available in 14 of 20 subjects. Main causes for failure were related to problems of the sensor needle dislodgment or the large dispersion of ICP values secondary to leakage of blood. We noticed that effects of needle dislodgment and blood leakage were more obvious in the intact group than the castration group. That may be because the intracavernosal pressure was higher (80 *vs* 30 mmHg) in rats with normal erectile function bearing ES-induced penis tumescence.

In groups with the penis being cannulated into the shaft, the needle was inserted along the longitudinal axis of the corpus cavernosum at the midpoint of the penile shaft. This allowed for easier and deeper cannulation. It also allowed for further adjustment of the needle. Usually, 6–8mm cannulation depths was attained by this method. The needle hardly dropped out of the penile shaft and blood leakage rarely occurred. This method was completed successfully in all of the 20 rats. Moreover, we obtained similar ICP values using both cannulation approaches in the available rats.

Beside comparisons of the two cannulation sites, some unique features of our experiment are worth mentioning: (1) We established a uniform protocol for the ES by 2.5 V with 15 Hz at 5 ms in a 60 second cycle and having the cycle repeated every five minute for three times in our study. This parameter was selected by examining the erectile responses of CN to ES at different voltages. We observed that 2.5V was the threshold stimulus for the induction of erection in both the normal and the castrated rats. Erectile response could not be evoked below 2.5V. The ICP was gradually rising but not statistically from 2.5V to 10V. And we found that the CN would appear to suffer electric burn and the rat would quiver violently as in electrical shock when the ES voltage increased above 10V. Hence, we chose 2.5V in all subsequent experiments. We also evaluated the effect of different frequencies at 10, 15 and 20 Hz, with 2.5 V. No significant differences were observed. Yet, the ES parameters of the ICP measurement may vary somewhat between laboratories. Different electrical voltages (ranging from 0.75 V to 7.5 V [13, 30, 31]) and frequencies (ranging from 1.5 Hz to 32 Hz [32–34]) have been used to stimulate the CN. Due to other investigators with particular stimulator equipment, it is acceptable to perform the ICP assessment applying inconsistent ES parameters. (2) The MPG located posterolaterally to the dorsal lobe of the prostate, but it may not be easily identified for inexperienced investigators. During the experiment, we observed that the MPG could be readily identified at its location near the internal iliac vein. Thus, we used the internal iliac vein as an anatomic landmark to find the MPG, then the CN arising from the MPG could be found subsequently. (3) The bipolar hook should not be placed on the MPG. Both our study and Cellek’s study [35] found that stimulation of the MPG would cause contraction of the bladder, pelvic floor and rectum. This would result in an inaccurate measurement of CN-mediated neurotransmissions, as the CN is the main innervation responsible for the erection [36]. (4) In the method of cannulation into the shaft of the penis, the cannulating needle should enter the penile shaft at about a 10-degree angle. According to our experience, more exaggerated angulation would result in perforating the urethra or entering the contralateral side of the cavernosum. (5) We tended to place an insulator between the positive electrode and negative electrode to prevent short-circuiting in the bipolar hooks. Otherwise, the electrodes would be easily in contact with each other owing to their close proximity. In addition, we encased a piece of latex glove at the distal end of electrical stimulators to prevent stimulation of the surrounding tissues (like striated abdominal muscle). (6) We routinely injected 50 microliters of heparinized saline solution into the corpora cavernosum. This proved to be an effective means of preventing the cavernosal tissue from clotting and affecting the pressure conductivity.

The potential limitation of this study is that both cannulation methods are an invasive procedure. Since corpus cavernosal tissue section mostly derived from the shaft of the penis rather than the crus of the penis, it is noteworthy that cannulation of penile shaft might damage the corpus cavernosal tissue and adversely affect its histological examination (i.e. hematoxylin-eosin staining, Masson’s trichrome staining, and immunohistochemistry).

## Conclusion

The optimal erectile response by ES of the CN in rats was found to be 2.5 V at a frequency of 15 Hz in the present study. The method of cannulation into the penile shaft for ICP assessment provided a significantly higher success rate compared to the method using the cannulation of the penile crus. The method of cannulating into the shaft the penis appears to serve as a better alternative for experimental application of the ICP measurement in rats.

## Supporting information

S1 Video Real-time displays of the intracavernosal pressure (ICP) measurement by the method of cannulation into the penile shaft. First, electrical-stimulation of the cavernosal nerve caused the tumescence of the cavernosum and induced erection. Then, the changes of intracavernosal pressure (ICP) were recorded by the analysis software of AcqKnowledge 4.2.1. Last, the penile began detumescent and the peak ICP fell to the baseline in the AcqKnowledge when the electrical-stimulation discontinued.
(MP4)

S1 Fig Blood reflux in the piping system after cannulation. (TIF)

S1 Table The ARRIVE (Animal Research: Reporting In Vivo Experiments) guidelines checklist. (DOCX)

S2 Table Laboratory protocols of the procedure of ICP measurement in protocols.io. (PDF)

S3 Table Changes in body weight in the intact group and the castration group. (DOC)

S1 File Raw research data. (XLSX)
