# Supplementary figures and images for: Comparison of two cannulation methods for assessment of intracavernosal pressure in a rat model
Source: PLoS One. 2018 Feb 27;13(2):e0193543. doi: 10.1371/journal.pone.0193543 (PMC5828359; doi:10.1371/journal.pone.0193543)

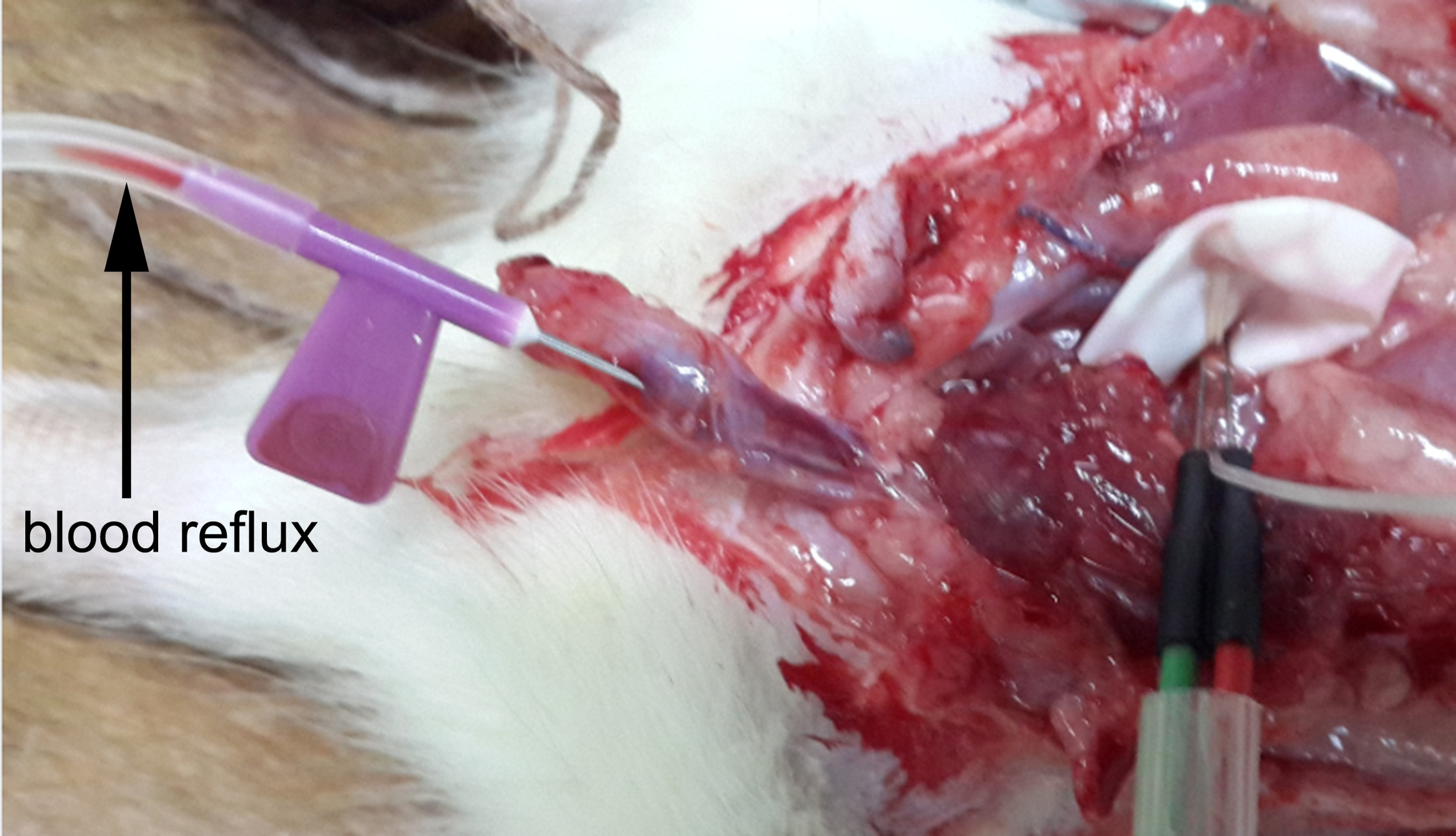

Supplement: S1 Fig — (TIF) [file pone.0193543.s002.tif]
