# Supplementary material for: Comparison of two cannulation methods for assessment of intracavernosal pressure in a rat model
Source: PLoS One. 2018 Feb 27;13(2):e0193543. doi: 10.1371/journal.pone.0193543 (PMC5828359; doi:10.1371/journal.pone.0193543)

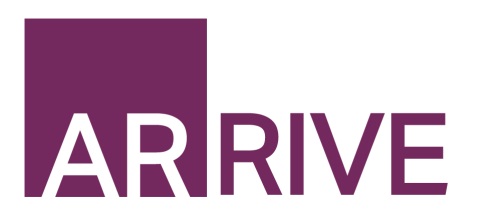


The ARRIVE Guidelines Checklist

Animal Research: Reporting In Vivo Experiments

Carol Kilkenny^1^, William J Browne^2^, Innes C Cuthill^3^, Michael Emerson^4^ and Douglas G Altman^5^

*^1^The National Centre for the Replacement, Refinement and Reduction of Animals in Research, London, UK, ^2^School of Veterinary Science, University of Bristol, Bristol, UK, ^3^School of Biological Sciences, University of Bristol, Bristol, UK, ^4^National Heart and Lung Institute, Imperial College London, UK, ^5^Centre for Statistics in Medicine, University of Oxford, Oxford, UK.*

|  | | ITEM | RECOMMENDATION | Section/ Paragraph |
| --- | --- | --- | --- | --- |
| 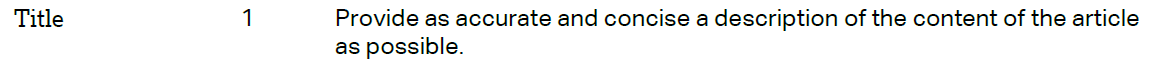 | | | Title |  |
| 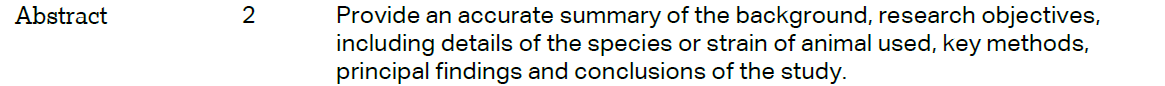 | | | Abstract |  |
| INTRODUCTION | | |  |  |
| 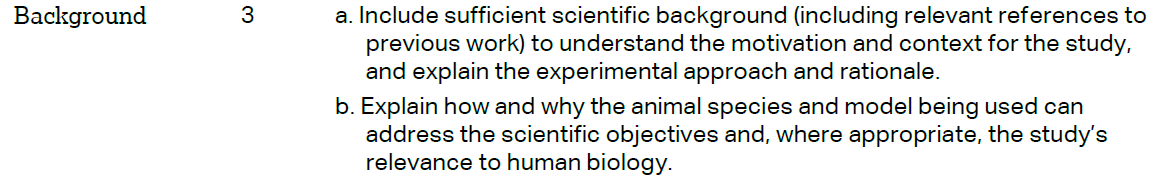 | | | Paragraphs 1-3  Paragraphs 2,3 |  |
| 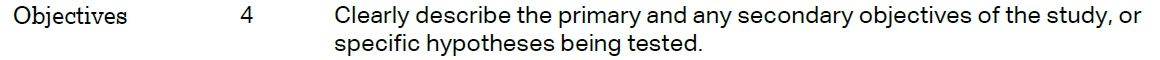 | | | Paragraphs 3 |  |
| METHODS | | |  |  |
| 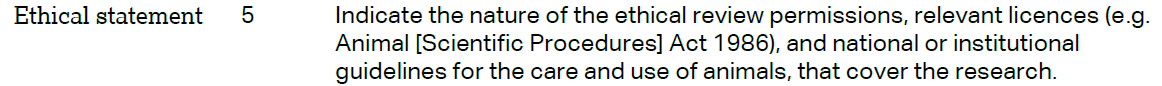 | | | Methods  Paragraphs 2 |  |
| 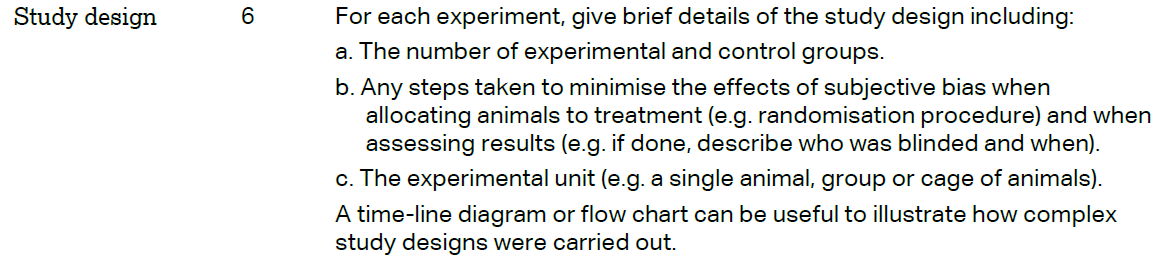 | | | Paragraphs 1,3  Paragraphs 1,4  Paragraphs 1,3,4 |  |
| 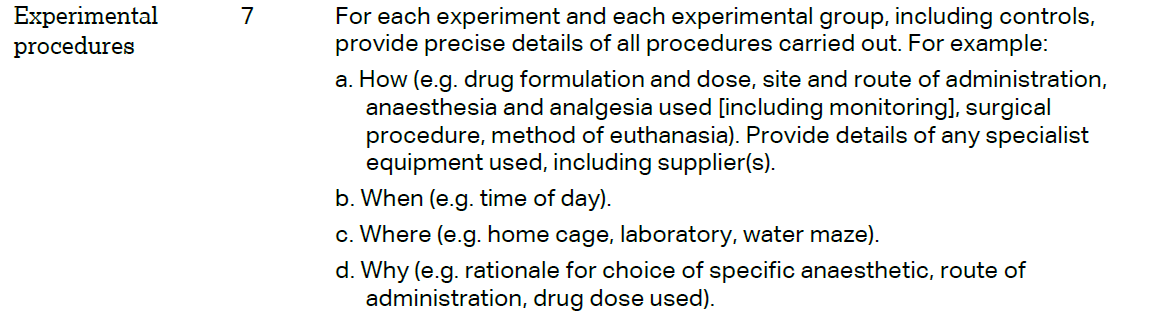 | | | Paragraphs 1,4-9 |  |
| 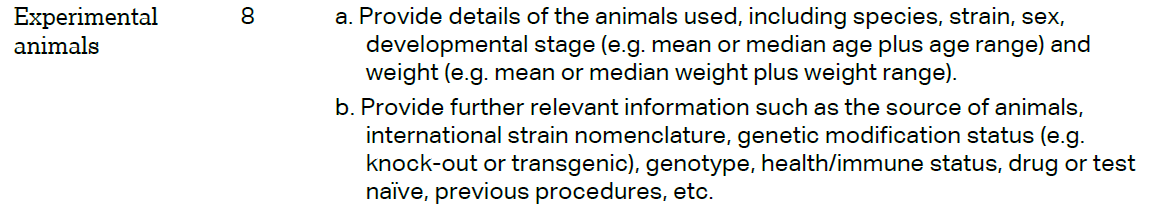 | | | Paragraphs 1 |  |

The ARRIVE guidelines. Originally published in *PLoS Biology*, June 2010^1^

| 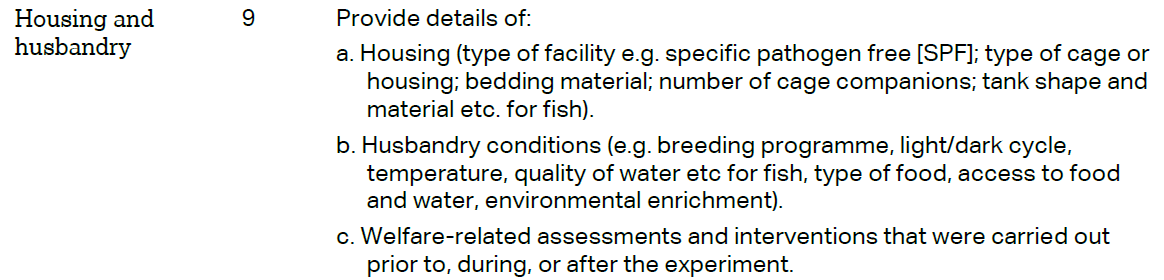 | Paragraphs 2 | |
| --- | --- | --- |
| 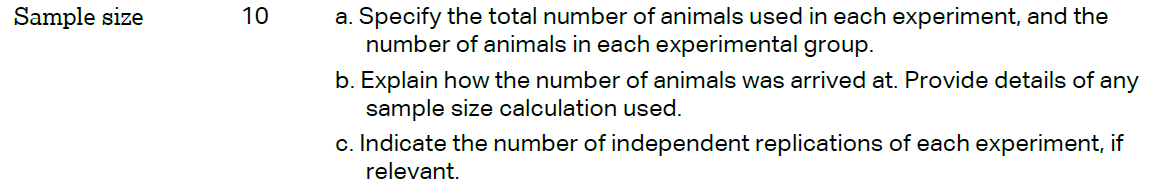 | Paragraphs 1,3,4  The sample size used in this study was based on previous studies. PMID: 19338748; 18668142;19453894. | |
| 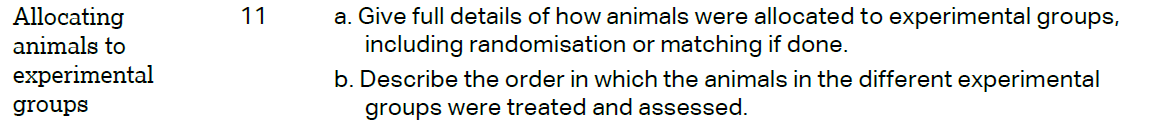 | Paragraphs 1 | |
| 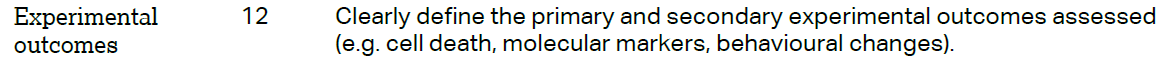 | Paragraphs 4,8,9 | |
| 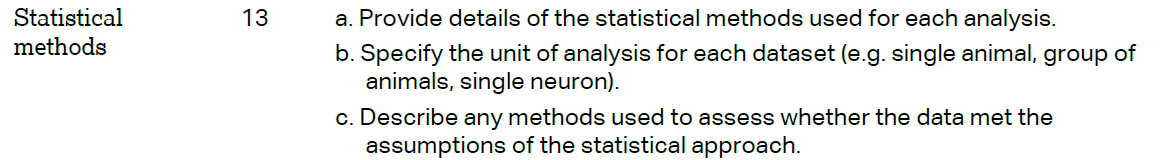 | Paragraphs 10 | |
| RESULTS |  | |
| 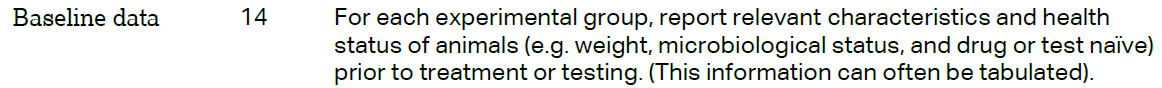 | Results  Paragraphs 1 | |
| 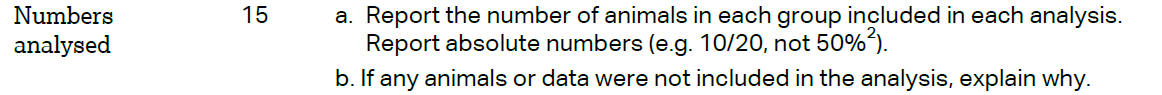 | Paragraphs 3,4 and Table 1 | |
| 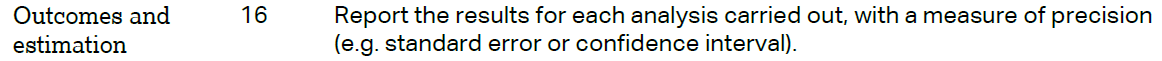 | Paragraphs 2-4;Table 2 and Figure 4,5 | |
| 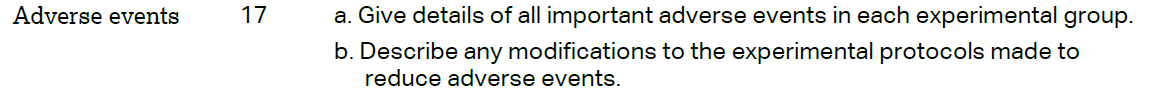 | Paragraphs 3,4 | |
| DISCUSSION |  | |
| 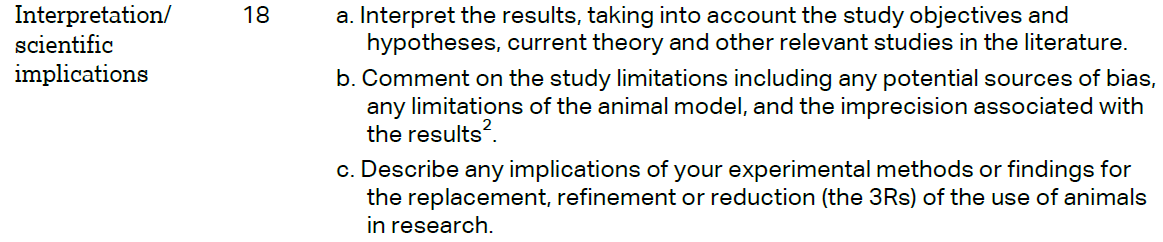 | Introduction  Paragraphs 1  Discussion  Paragraph 2，3  Paragraphs 4,5 | |
| 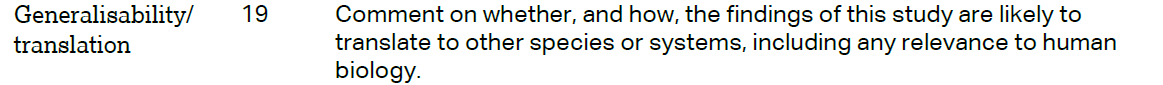 | None | |
| 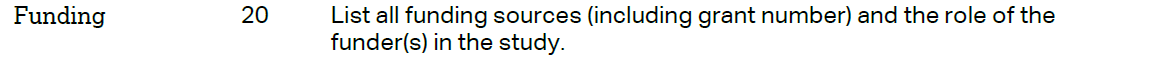 | | None |


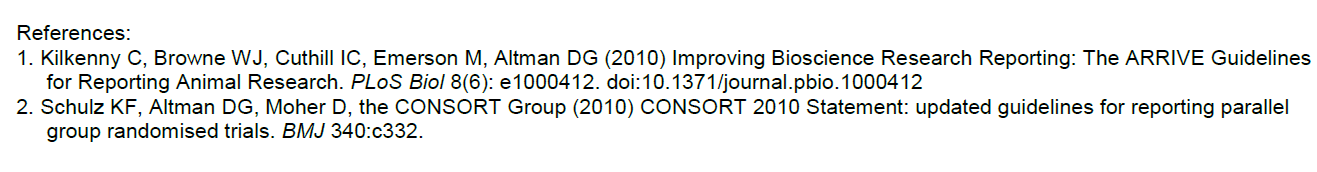

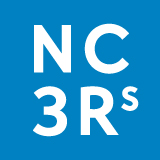

Supplement: S1 Table — (DOCX) [file pone.0193543.s003.docx]
