# Supplementary material for: Comparison of two cannulation methods for assessment of intracavernosal pressure in a rat model
Source: PLoS One. 2018 Feb 27;13(2):e0193543. doi: 10.1371/journal.pone.0193543 (PMC5828359; doi:10.1371/journal.pone.0193543)
Supplement: S2 Table — (PDF) [file pone.0193543.s004.pdf]

A protocol for assessment of intracavernosal pressure in a rat model

Shankun Zhao,Ran Kang,Zhigang Zhao

Abstract

Intracavernous pressure (ICP) measurement is a well-established technique for assessing the erectile function, which was performed by cannulating either crus or shaft of the penis. But yet, as far as we know, no consensus has reached on the ES parameters of frequency and voltage response. Moreover, the current technique for ICP measurement in the rat is difficult to perform, especially in the cannulation of the corpus cavernosum. It often leads to failure in the experiment and takes time to explore the optimum conditions for inexperienced investigators. In present study, we provided a detailed step-by-step procedure of ICP measurement.

Citation: Shankun Zhao,Ran Kang,Zhigang ZhaoA protocol for assessment of intracavernosal pressure in a rat model. **protocols.io**
dx.doi.org/10.17504/protocols.io.kwdcxa6
Published: 22 Nov 2017

Protocol

Different voltages and frequencies on electrical stimulation (ES) parameters

1. The ES equipment was composed of a bipolar hook electrode and an integrated data acquisition system (MP 150A-CE; Biopac, Santa Barbara, CA). All data were analyzed using the AcqKnowledge software, version 4.2.1 (Biopac System Inc). The application of different voltages and frequencies were used in the current protocol to achieve a significant and consistent erectile response. Under anaesthetic (pentobarbital, 45 mg/kg), rats were firstly subjected to sequential stimulation strengths ranging from 1 to 10 volts at a frequency of 15 Hz with a square pulse wave form and a duration of 5 milliseconds for 60 seconds. The optimal voltage parameter according to the voltage response curves was logged. Next, the effect of different frequencies (10, 15 and 20 Hz) at the selected voltage that resulted in the highest erectile response was assessed. Three ESs were replicated at intervals of 5 min.

Mean arterial pressure (MAP) measurement

2. The right carotid artery was exposed and the cephalad end of the carotid artery was ligated by a 4-0 silk suture. This would prevent obscuring of the visual field from the leakage of blood. Next, a slipknot with a silk suture was tied around the proximal end carotid artery. A 24-G angiocatheter filled with heparin and connected to the pressure transducer was then inserted caudally into the carotid artery. Afterwards, the slipknot suture was tightened to prevent the sensor needle falling out of the vessel. The ratio between the ICP and MAP was calculated to normalize the variations in systemic blood pressure.

Exposure of the cavernous nerve (CN) and fixation of the electrode

3. By retracting the intestine, the seminal vesicles and the bladder laterally, the fascial layer covering the prostate could be exposed posteriorly simply using cotton swabs. We had noted that the prostate gland was usually atrophic in the castrated rat; it was thus often difficult to identify. The star-like MPG, located posterolaterally and symmetrically to the dorsal lobe of the prostate, was visualized. At this time, the internal iliac vein could be seen directly adjacent to the dorsal lobe of the prostate, following its curvature. This is an anatomic landmark we use for identifying the MPG. The MPG divides into three major branches. The largest that runs along the surface of the membranous urethra is the CN. The pelvic nerve is situated inferior to the MPG. And the proximal branch that disperses along the intraabdominal position is the hypogastric nerve. We preferred to do the isolation of the CN using a glass needle. Next, a tiny stainless steel bipolar hook electrode with a diameter of 0.3 millimeters was placed around the CN, 24 millimeters distal to the MPG, for ES. The positive electrode was placed proximal to the MPG and the negative electrode at the distal end. To prevent shorting circuits, the parallel hooks of the electric stimulator were separated from each other by an insulator with a one-millimeter diameter. In addition, we recommended encasing a piece of latex glove to isolate the ends of the electrical stimulators in order to prevent the ES of the surrounding tissues.

This is an open access protocol distributed under the terms of the Creative Commons Attribution License, which permits unrestricted use, distribution, and reproduction in any medium, provided the original author and source are credited

Cannulated the corpus cavernosum

4. After the bipolar electrodes were in place, the penis was degloved and the penile ischiocavernous muscle was divided to one side by blunt dissection. This allowed entry into the underlying tunica albuginea of the crus of the corpus cavernosum. A 24-gauge venoclysis needle was inserted into this side of crus (for cannulating in penile shaft: the needle was inserted into the midpoint of the corpus cavernosum) for the ICP measurement. 50 microliters of heparinized saline solution (250 U/ ml) were injected into the corpora cavernosum with a syringe through the T-shaped connection pipe to prevent clotting of the needle tip.

Monitoring ICP

5. Basal ICP was firstly assessed using the analysis software of AcqKnowledge 4.2.1. The neurostimulation of the CN was next performed and the changes of ICP were recorded. The mean maximum ICP and the total ICP of the tumescence determined by the AUC from the beginning to the end of the CN stimulation (60 seconds) were recorded. The ES was always done in triplets with a 5-minute interval between the subsequent stimulations to ensure stable activity in every rat.

This is an open access protocol distributed under the terms of the Creative Commons Attribution License, which permits unrestricted use, distribution, and reproduction in any medium, provided the original author and source are credited
