# Supplementary material for: Comparison of two cannulation methods for assessment of intracavernosal pressure in a rat model
Source: PLoS One. 2018 Feb 27;13(2):e0193543. doi: 10.1371/journal.pone.0193543 (PMC5828359; doi:10.1371/journal.pone.0193543)
Supplement: S3 Table — (DOC) [file pone.0193543.s005.doc]

**Supplementary Table 3.** Changes of body weight in the intact group and the castration group.

| Group | n | Body weight (g) | |
| --- | --- | --- | --- |
|  |  | initial | 4 weeks |
| intact group | 25 | 336.4±10.19 | 454.2±14.88 |
| castration group | 25 | 340.1±12.60 | 407.3±9.51 |
| *t* |  | -1.147 | 13.287 |
| *P* |  | 0.257 | 0.000 |

Values are expressed as the mean ± SEM. Comparisons between two groups using a Student’s *t-*test.
